# Dilemmas of early dementia screening among community-dwelling older adults: a qualitative study from the perspective of social determinants of health

**DOI:** 10.3389/fpubh.2026.1927041

**Published:** 2026-09-16

**Authors:** Lining Xi, Junli Wan, Qifang Liu, Jingyan Liu, Huidan Yu, Xianwu Luo

**Affiliations:** School of Nursing, Wuhan University, Wuhan, Hubei, China

**Keywords:** community-dwelling older adults, dementia, early screening, qualitative study, social determinants of health, thematic analysis

## Abstract

**Background:**

Dementia is one of the most urgent public health challenges globally. Early screening is indispensable for timely identification and intervention. However, early dementia screening among community-dwelling older adults remains low, and the mechanisms underlying screening barriers remain insufficiently understood.

**Methods:**

A qualitative study involving semi-structured interviews was conducted with 23 older adults who were purposively recruited from three communities in Wuhan, China. Interviews were transcribed verbatim and analyzed using thematic analysis that combined deductive coding guided by the social determinants of health framework with inductive coding to capture themes emerging from the data.

**Results:**

Five themes and 12 subthemes were identified: (1) economic stability, comprising economic burden concerns and the lack of material incentives; (2) education access and quality, comprising limited disease-related knowledge, the double effect of educational level, and fixed beliefs about dementia risk; (3) social and community context, comprising perceived stigma, result-related anxiety and family concerns, and late-life values; (4) health care access and quality, comprising insufficient medical trust and service assurance, limited information accessibility, and symptom-oriented healthcare-seeking behavior; and (5) neighborhood and built environment, comprising limitations in convenience and accessibility.

**Conclusion:**

Low uptake of early dementia screening among community-dwelling older adults in China reflects a system-level behavioral dilemma shaped by social determinants of health. Future screening programs should build integrated, community-embedded pathways that make screening affordable, understandable, trustworthy, connected, and convenient, thereby strengthening the acceptability, equity, and sustainability of dementia prevention and early detection.

## 1 Introduction

Dementia is one of the most pressing global public health challenges (1). China has the world’s largest population of people living with dementia, with an estimated 12.1 million adults aged 50 years or older affected in 2020; this number is projected to reach 66.3 million, accounting for 10.4% of this age group, by 2050 (2). Dementia progressively impairs not only the cognitive function, daily activity capacity and independence of affected individuals, but also imposes substantial burdens on families, healthcare systems, and society (1, 2). Dementia-related socioeconomic costs in China are projected to reach US$114.2 billion by 2030 (1).

Early screening and intervention are core strategies for mitigating this burden (3). The World Health Organization’s Global Action Plan on the Public Health Response to Dementia 2017–2025 emphasizes community- and primary care-based approaches to delay disease progression, reduce complications, and lower societal costs (4). In late 2024, the National Health Commission launched an initiative to establish an integrated system for prevention, screening, early intervention, and standardized care by 2030 (5).

Despite these policy commitments, dementia screening remains difficult to implement in community settings. A recent survey in Wuhan found that only 17.4% of older adults in urban communities had received dementia screening or cognitive testing in the previous year (6). This rate remains far below the national coverage target of 80% (7). In many regions, primary care diagnosis rates remain below 10% (8, 9). These figures underscore a marked gap between policy ambitions and implementation.

Previous research has identified multiple barriers to dementia screening and management in China (8). These include limited knowledge among primary care providers, unclear referral pathways (8), constrained consultation time (10), low disease awareness among patients and families (11), and concerns about stigma (12) and caring burden (13). Most studies have examined these barriers primarily at the individual, family, or community level (14). Broader social determinants of screening decisions have received less attention (8, 15). In particular, qualitative evidence capturing the perspectives and everyday realities of community-dwelling older adults remains scarce (6).

The social determinants of health (SDOH) framework offers a comprehensive lens for understanding these challenges (16). SDOH highlights how economic stability, education, social support, and access to healthcare systematically shape health behaviors and outcomes (17). In China, these determinants intersect with cultural and social features, including filial piety, dense social networks, and rapid urbanization (18). These intersections generate multilayered barriers that have rarely been examined in an integrated way (19). Although SDOH perspectives are increasingly used in global dementia research (15), context-specific qualitative evidence on the everyday realities of older adults in China remains limited. A few previous studies have focused on isolated SDOH domains or used quantitative designs (18), offering limited insight into culturally embedded mechanisms.

This qualitative study addresses this gap. We systematically examine barriers to early dementia screening among community-dwelling older adults through an SDOH lens. Drawing on participants’ perspectives, we examine how family, community, and societal factors interact to shape screening decisions in contemporary China. These barriers are shaped by both structural conditions and cultural norms. By uncovering these dynamic interactions, this study provides nuanced insight into barriers to screening. It also provides empirical evidence to inform more equitable and person-centered strategies for dementia prevention and care.

## 2 Materials and methods

### 2.1 Study design

We conducted a descriptive qualitative study to examine structural barriers to early dementia screening among community-dwelling older adults through the lens of the social determinants of health (SDOH) (20). Thematic analysis was used to identify, interpret, and report recurring patterns in the data (21). The SDOH framework informed the study design, interview guide, and data analysis, enabling a multilevel examination of how individual, community, and societal factors interact to shape dementia screening barriers in China.

### 2.2 Sampling and participants

Participants were purposively recruited from three communities in Wuhan, China. To capture variation in SDOH contexts, three administrative districts were selected based on socioeconomic, social, healthcare, and geographic characteristics, with one community randomly selected from each district. Older adults approached through community health services were asked about their willingness to undergo dementia screening; those expressing unwillingness or hesitation and meeting the eligibility criteria were considered for inclusion. Maximum variation sampling was used to ensure diversity in age, education, income, living arrangements, occupation, prior exposure to people with dementia, and community context.

The inclusion criteria were as follows: (1) age ≥60 years; (2) residence in the community for at least 1 year; (3) ability to understand and communicate verbally; and (4) no apparent severe cognitive impairment that would preclude completion of the interview. The exclusion criteria were as follows: (1) a confirmed history of dementia or other severe cognitive impairment; (2) severe hearing, speech, or psychiatric disorders that prevented completion of the interview; (3) an acute cerebrovascular event or other serious acute illness within the preceding 3 months. Recruitment continued until data saturation was reached, defined as the point at which no new themes or subthemes emerged from additional interviews.

### 2.3 Data collection

Data were collected between September 2025 and May 2026 through semi-structured, in-depth interviews. The interview guide was informed by the five core SDOH domains and a review of relevant literature. The guide was reviewed by two nursing experts and one public health expert, pilot-tested with two older adults, and refined accordingly. Core questions included: “How do you perceive early dementia screening?,” “What factors influence your decision to participate in dementia screening?,” and “How might a positive screening result affect you and your family?” These questions were designed to elicit open accounts of participants’ experiences and perceived barriers.

All interviews were conducted by the main researcher, who had received training in qualitative research methods. To protect privacy and encourage candid discussion, interviews were conducted in participants’ homes or quiet community rooms and lasted approximately 40–60 min. Before each interview, the researchers explained the study aims, procedures, voluntary nature of participation, right to withdraw, and anonymization measures, and obtained written informed consent. All interviews were audio-recorded with participants’ permission.

Data collection and preliminary analysis proceeded concurrently. Emerging themes and subthemes were reviewed by two researchers throughout data collection. No new themes or subthemes emerged after the 21st interview, and two additional interviews were conducted to confirm data saturation, resulting in a final sample of 23 participants.

Another researcher documented non-verbal cues, including emotional responses and body language, in field notes. The main researcher transcribed all recordings verbatim within 24 h. Transcript accuracy was independently verified by another researcher. Reporting followed the Consolidated Criteria for Reporting Qualitative Research (COREQ) (22).

### 2.4 Data analysis

We analyzed the data using Braun and Clarke’s six-phase approach to thematic analysis (21), combining deductive and inductive coding. The SDOH framework served as the primary deductive lens, with its five domains guiding the initial coding structure. This enabled systematic identification of multilevel influences. Inductive coding was also used to capture themes and insights that extended beyond the predefined SDOH domains.

Two researchers independently read the transcripts several times to become familiar with the data before generating initial codes. Codes were then mapped to SDOH domains or developed into new subthemes. Regular team discussions were held to assess thematic coherence, resolve coding discrepancies by consensus, and refine theme definitions and labels. We specifically examined cross-domain interactions to understand how different SDOH elements jointly shaped screening barriers. NVivo 12.0 was used to organize the data and support coding. The final coding framework was iteratively refined and finalized through team discussion.

### 2.5 Ethical considerations

The study was approved by the Life Sciences and Medical Ethics Committee of Wuhan University (Approval No. WHU-LFMD-IRB2026022). All procedures followed the Declaration of Helsinki for human biomedical research (23). All participants were fully informed about the study and provided written informed consent, with assurances of confidentiality, voluntary participation, and the right to withdraw at any time without penalty. Participant privacy was protected through anonymization, encrypted data storage, and restricted access. Audio recordings and transcripts will be destroyed 5 years after completion of the study.

### 2.6 Rigor

To ensure rigor, we followed Lincoln and Guba’s criteria for trustworthiness: credibility, dependability, confirmability, and transferability (24). Both researchers had expertise in public health and cognitive health, prior experience interviewing older adults, and additional training in qualitative methods before data collection. The interview guide was developed from a comprehensive review of the literature and refined through expert review and pilot testing.

Reflexivity was supported through regular team meetings in which researchers discussed their assumptions and their potential influence on data interpretation. To enhance the credibility and confirmability of the findings, summarized interview information was returned to selected participants to verify whether the accounts accurately reflected their views and experiences. An audit trail was also maintained, including audio recordings, verbatim transcripts, coding records, analytic memos, and documentation of theme development, allowing interpretations to be traced back to the original data. These procedures helped ensure that the findings accurately represented participants’ perspectives and remained grounded in the data.

## 3 Results

A total of 23 community-dwelling older adults participated in the study, including 9 men and 14 women, with a mean age of 72.00 ± 9.52 years. Participants varied in educational attainment, monthly income, living arrangements, former occupation, and prior exposure to people with dementia, reflecting diversity in sociodemographic and experiential characteristics relevant to SDOH. Participants’ sociodemographic characteristics are summarized in Table 1.

**Table 1:** Participant demographic characteristics (*N* = 23).

Participant ID	Age	Gender	Education level	Living situation	Economic status (monthly income, CNY)	Presence of dementia around	Pre-retirement occupation
P1	65	Female	High school	Living with children	≥9,000	Yes	Enterprise unit
P2	62	Female	Associate degree or above	Living with spouse	6,000–8,999	No	Enterprise unit
P3	64	Male	High school	Living with spouse	<3,000	No	Driver
P4	62	Female	High school	Living alone	3,000–5,999	No	Worker
P5	72	Female	Middle school	Living with children	3,000–5,999	No	Worker
P6	80	Male	Associate degree or above	Living alone	3,000–5,999	No	Government/public institution
P7	65	Male	Middle school	Living with spouse and children	3,000–5,999	No	Worker
P8	68	Male	High school	Living with spouse	3,000–5,999	No	Government/public institution
P9	71	Male	Middle school	Living with spouse	6,000–8,999	No	Enterprise unit
P10	61	Male	Associate degree or above	Living with spouse and children	6,000–8,999	No	Enterprise unit
P11	72	Female	Primary school or below	Living with children	<3,000	No	Farmer
P12	60	Female	Primary school or below	Living alone	<3,000	No	Farmer
P13	62	Female	Middle school	Living with spouse	3,000–5,999	No	Worker
P14	64	Female	High school	Living alone	3,000–5,999	No	Worker
P15	74	Female	Middle school	Living with spouse	3,000–5,999	No	Enterprise unit
P16	74	Male	Middle school	Living with spouse	<3,000	No	Worker
P17	76	Female	Middle school	Living with spouse	3,000–5,999	No	Worker
P18	76	Female	Middle school	Living with sister	3,000–5,999	No	Government/public institution
P19	96	Male	Associate degree or above	Living alone	≥9,000	No	Government/public institution
P20	82	Female	Associate degree or above	Living alone	≥9,000	No	Government/public institution
P21	84	Female	High school	Living alone	6,000–8,999	No	Government/public institution
P22	86	Male	Associate degree or above	Living with spouse	≥9,000	No	Worker
P23	80	Female	Middle school	Living with spouse	3,000–5,999	Yes	Government/public institution

### 3.1 Results of the directed analysis

Guided by the social determinants of health (SDOH) framework, thematic analysis identified five overarching themes and 12 subthemes describing barriers to participation in early dementia screening. The five themes were economic stability, education access and quality, social and community context, health care access and quality, and neighborhood and built environment. Figure 1 illustrates the thematic structure of barriers faced by community-dwelling older adults in early dementia screening through an SDOH lens.

**Figure 1:**
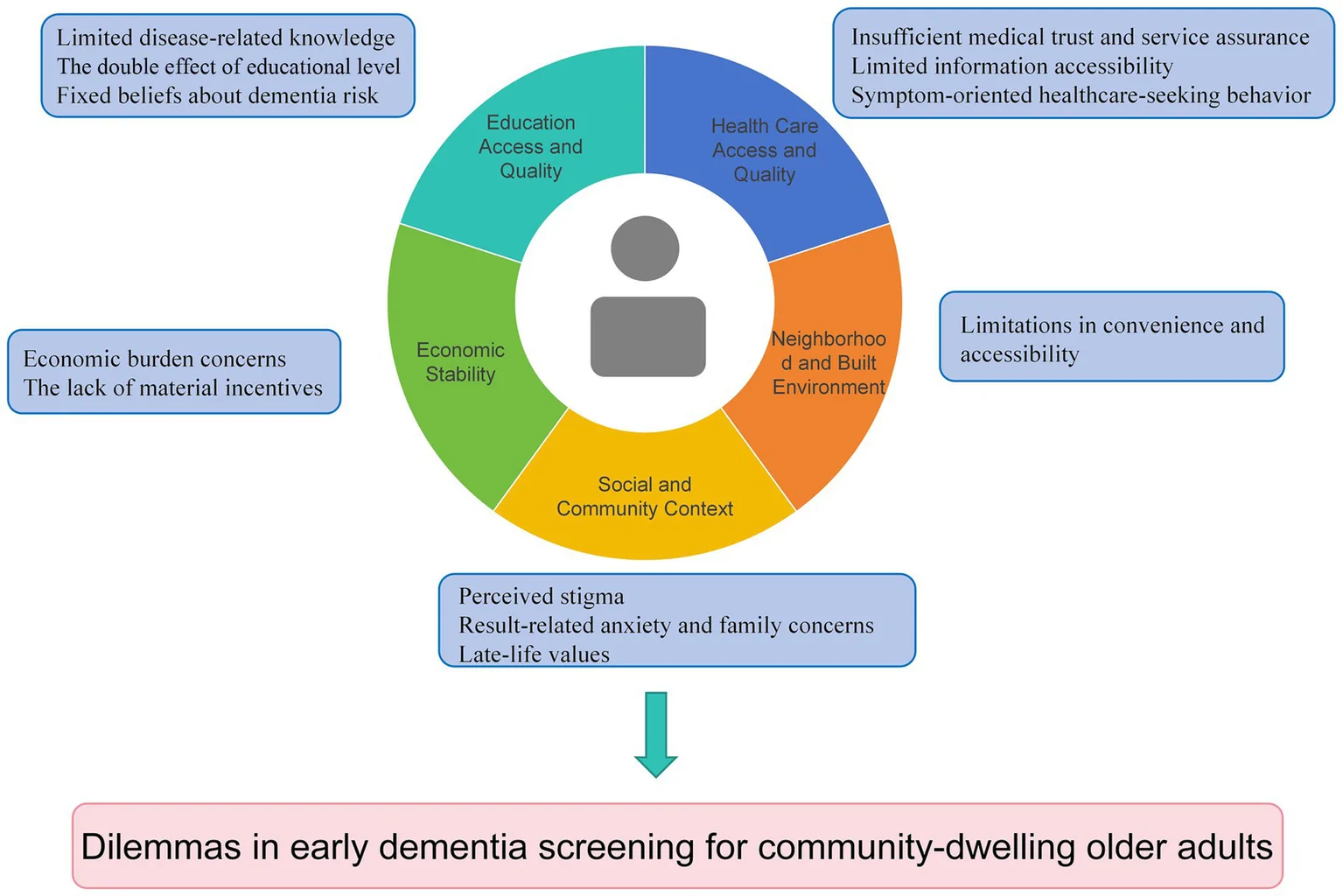
Dilemmas in early dementia screening for community-dwelling older adults from a social determinants of health (SDOH) perspective.

### 3.2 Theme 1: economic stability

Economic stability shaped how older adults weighed the affordability and perceived value of dementia screening. Concerns about screening and follow-up care costs were intertwined with questions about whether participation justified the time and effort involved. Economic stability therefore operated not simply as an issue of payment capacity, but as a practical condition shaping screening willingness.

#### 3.2.1 Economic burden concerns

For most participants, payment was the first concern when considering dementia screening. Self-payment, particularly a high out-of-pocket share, reduced willingness to participate. Even modest costs mattered, as insurance coverage and personal payment shaped screening decisions.

*“If I have to pay out of pocket, the burden is a bit heavy… Paying about 30% out of my own pocket feels too much for me. It would be best if I only had to pay within 10% out of pocket” (P2).*

*“If it requires self-payment, I would not take part; I do not want to pay anything at all.” (P16).*

Economic concerns extend beyond the cost of screening itself. An abnormal result is considered likely to trigger additional expenses for diagnosis, treatment, medication, long-term care, and institutional care. For participants who have limited pensions or worry about becoming a burden to their children, forgoing screening becomes a strategy to avoid expected financial obligations.

*“What difference would it make if screening found something? If I had to go to a nursing home… a retired worker only gets just over 3,000 yuan a month. How could I afford that?” (P18).*

*“If screening showed dementia and it cost money, my children would definitely worry. They have their own children and families to support. Even regular hospital tests already cost a lot; if dementia brought bigger expenses, they could not handle it.” (P11).*

#### 3.2.2 The lack of material incentives

Beyond economic burden, several participants noted that small gifts or benefit-oriented arrangements could increase willingness to participate in screening. Rather than reflecting purely material motivation, this suggested that external incentives could trigger participation when screening value was not fully understood or its health benefits were not immediately visible. For older adults who perceived themselves as healthy and felt little urgency, material incentives offered an immediate sense of return and increased the likelihood of initial engagement.

*“They might be more willing if you give them something small for coming. Without that, they think they are healthy and do not want to do any checks.” (P3).*

*“Even a small gift would make them more likely to join. They may not care much what the activity is; as long as there’s something to receive, they feel their effort has paid off right away.” (P4).*

### 3.3 Theme 2: education access and quality

Education access and quality shaped older adults’ understanding of dementia risk and their assessment of the necessity of screening. This study suggests that limited understanding of dementia and dementia screening was not merely a function of educational attainment, but was embedded in how participants interpreted disease risk, early cognitive changes, and the value of professional screening.

#### 3.3.1 Limited disease-related knowledge

Most participants understood dementia primarily as severe functional decline occurring in middle- or late-stage disease, with little awareness of the clinical significance of early cognitive changes. Cognitive decline was generally regarded as a result of constitution, fate, heredity, or normal aging, rather than a risk signal that requires clinical assessment. Mild memory impairment and daily forgetfulness were rarely associated with early detection, which reduced people’s perceived demand for dementia screening.

*“I still think screening does not really change anything. Getting this disease depends on your constitution. If you are meant to get it, you cannot avoid it; if you are not, you will not.” (P12).*

*“My parents were very quick-minded, and everyone in my family is sharp. Genetically, we do not have that problem, so I do not need screening.” (P3).*

*“At this age, memory naturally gets worse. After 80, it becomes more obvious. Misplacing things and forgetting things happen all the time, but I think that’s normal. People are like that when they get old.” (P21).*

#### 3.3.2 The double effect of educational level

Educational attainment shaped screening willingness through two contrasting pathways. Lower educational attainment was associated with limited understanding of dementia and screening value, whereas higher education or professional experience sometimes fostered reliance on self-judgment and confidence in managing cognitive changes independently. Despite different mechanisms, both pathways weakened motivation for early screening.

*“People with little education, or those who cannot read, may know less about this and think it does not really matter.” (P4).*

*“People with higher education may actually be less willing to be screened. Like me, I know a little about the relevant process and what steps to do, so I do not think I need anyone else to do the screening for me.” (P22).*

*“With my education and professional knowledge, I do not think I need screening.” (P20).*

#### 3.3.3 Fixed beliefs about dementia risk

Some participants held fixed beliefs about dementia risk, viewing dementia as distant and personally unlikely on the basis of their perceived health. These fixed beliefs encouraged reliance on personal experience and self-assessment rather than external screening results. Accordingly, screening was seen less as a method of detecting early risk, and more as an unnecessary challenge to their existing understanding of their own health. In turn, this reduced the perceived necessity and urgency of screening.

*“I’m very sure of myself. I do not feel old at all… I do not believe I’ll ever get to that point. I trust myself. No matter what you tell me, I know my own body, and I believe my mind will stay clear until the day I die.” (P13).*

*“I’m cheerful, so how could I get dementia? People get dementia because they think too much and keep problems in their head. I’m open-minded and not afraid of death, so I will not get it… I live a regular life and do not eat, drink, or mess around carelessly, so I will not develop it.” (P17).*

### 3.4 Theme 3: social and community context

Social and community contexts shaped screening as more than a medical assessment. Through stigma, result-related anxiety, family concerns, and late-life values, screening became associated with social judgement, family responsibility, and life-stage meaning. For some older adults, avoiding screening was a way to avoid anticipated social, familial, and psychological consequences.

#### 3.4.1 Perceived stigma

Some participants feared that screening would make their health status visible within close-knit community networks and expose them to judgement or gossip. In the context of dementia-related stigma, an abnormal result could signal diminished ability or loss of normal functioning, threatening their sense of face and social image.

*“In our community, everyone knows each other. If I really had it, people would find out right away, and some would talk behind my back or laugh at me.” (P5).*

*“I do not want to be screened. Some people score low and some score high. If I scored low, I’d feel embarrassed because people might laugh at me. They do not understand; they’d just think a low score means dementia.” (P9).*

#### 3.4.2 Result-related anxiety and family concerns

The possibility of an abnormal screening result created psychological pressure for some participants. Even mild abnormalities could evoke persistent worry, anxiety, and fears of future disability, especially when participants were uncertain about whether cognitive decline could be treated or managed. For these participants, avoiding screening helped preserve psychological peace and prevent result-related anxiety.

*“If they found even a small problem, I’d carry that burden all the time. Life would feel so tiring. And if it cannot be cured, would not I just live every day worrying and being afraid? I’d rather not know.” (P14).*

*“I know my memory is not good, but I do not want other people, especially outsiders, to make me feel worse. The more you know, the more you worry; knowing less can be more peaceful. Why should I let you tell me again? That would only upset me more.” (P6).*

For many participants, anxiety about an abnormal result extended to its implications for the family. Results were interpreted not only as personal health information, but also as potential triggers of new caregiving responsibilities. An abnormal finding implied care arrangements, medical visits, supervision, and disruption of family routines. Within traditional expectations of family responsibility, some older adults feared burdening their children or becoming a long-term care responsibility if they lost functional independence.

*“If I really got dementia, it would definitely burden my family. If I wandered off, they’d have to look for me everywhere. I do not want to trouble them. They already have children to care for, and I do not want to disrupt things.” (P16).*

*“If they knew I might get dementia later, they’d definitely worry and make extra arrangements. That would become their burden. As a parent, of course I worry about adding to it.” (P6).*

#### 3.4.3 Late-life values

For some participants in advanced age, screening was evaluated through a late-life perspective. Having lived what they considered a sufficiently complete life, they were less inclined to change their current living situation even if cognitive problems were detected. Without clear benefits for quality of life, screening carried little motivation for participation.

*“I’m already in my eighties. I’ve seen what I needed to see, and I’m satisfied with my children and grandchildren. I do not really want to live much longer, so I do not see the need to go through all those tests and treatments.” (P6).*

*“If I were 60, screening might make sense. But I’ve already lived to 80, and that’s good enough. What’s the point of more checks? At this age, this is the path we all have to take, so why put myself through that?” (P17).*

### 3.5 Theme 4: health care access and quality

Healthcare access shaped the pathway from screening availability to actual participation. Distrust in community-based screening, uncertainty about follow-up diagnostic and treatment support, limited health education, and symptom-oriented healthcare-seeking disrupted older adults’ movement from awareness to trust and proactive engagement.

#### 3.5.1 Insufficient medical trust and service assurance

Most participants questioned the credibility of community-based dementia screening. Large hospitals and specialists were seen as more trustworthy, while community institutions were perceived as lacking sufficient experience, expertise, and diagnostic resources. These perceptions undermined confidence in the reliability of community screening results.

*“I still trust big hospitals more. They have more experience, more specialists, and better facilities. I’m not very confident about community hospitals, so if the screening was done there, I’d probably doubt the results.” (P6).*

*“I’d wonder whether community screening is really accurate. They just ask you questions, and I’m not sure how professional they are. If it was done at a big hospital, I’d feel it was more reliable.” (P14).*

Beyond doubts about screening accuracy, some participants questioned whether an abnormal result would be followed by clear and effective support. Without continuity of care, including diagnostic confirmation, treatment, referral, and long-term management, screening was seen as merely identifying problems without offering tangible benefit. Perceived limitations in dementia treatment further weakened the value assigned to screening.

*“If the screening shows I might be developing dementia, and I go to the hospital to confirm it, can they actually treat it? As far as I know, there is not really any medicine that can reverse or stop it.” (P18).*

*“Dementia treatment is still quite limited. I feel these tests are more for collecting research data, and they do not really help me much personally.” (P6).*

#### 3.5.2 Limited information accessibility

Participants’ limited access to information kept dementia screening outside their routine health decision-making. With little prior exposure to screening-related information and insufficient community publicity or health education, they more often interpreted memory decline as natural aging than as a signal requiring assessment. This limited their awareness of screening services and weakened proactive participation.

*“This is the first time I’ve heard of dementia screening. Our community has never organized it, so many older people do not even know it exists and just think memory loss is part of ageing.” (P1).*

*“The community has never organized this kind of screening or told us about it. I did not even know it existed.” (P2).*

#### 3.5.3 Symptom-oriented healthcare-seeking behavior

Some participants base healthcare-seeking decisions mainly on perceived symptoms, considering examinations only when discomfort became obvious or daily life was affected. As early cognitive changes are often subtle and may not immediately disrupt daily functioning, older adults tended to underestimate their need for screening. This symptom-oriented pattern made dementia screening less likely to be viewed as a proactive behavior for prevention and early risk identification.

*“I’m not really interested in these screenings. My memory has not declined noticeably, and I do not like doing this kind of thing. If my memory really got worse, I might go to the hospital.” (P22).*

*“I’d only get screened if I felt something was wrong with my body. I’d take action only when I actually felt unwell.” (P2).*

### 3.6 Theme 5: neighborhood and built environment

Neighborhood and built environment shaped the translation of screening availability into participation. Older adults’ willingness was constrained not only by their acceptance of screening, but also by where services were delivered and how well they fit into daily routines and responsibilities. Screening that was disconnected from everyday settings could be declined because of its added burden, even when screening itself was not rejected.

#### 3.6.1 Limitations in convenience and accessibility

Most participants viewed screening that required a separate trip as an added burden of time and physical effort. For those of advanced age, with mobility limitations, or with caregiving responsibilities, participation was constrained by the fit between screening arrangements and everyday life. Screening was more acceptable when delivered close to home or embedded in routine health checks or community activities. Accessibility was therefore less a matter of distance alone than of reducing the time, physical, and practical demands of participation.

*“Most older people do not walk very well. They do not even want to go to the hospital, let alone make a special trip for screening.” (P21).*

*“If it was really convenient, I might answer a few questions. But if I had to go there specially, sign up, and wait in line, I probably would not go.” (P6).*

*“I have to go home every day to cook. My husband is not well and needs help with toileting. My children all work, and I’m responsible for three meals a day… Who has time to make a special trip for screening?” (P11).*

## 4 Discussion

Guided by the social determinants of health (SDOH) framework (25, 26), this study examined barriers to early dementia screening among community-dwelling older adults (27, 28). Low screening willingness stems from systemic barriers across five interrelated SDOH dimensions: economic stability, educational opportunity and literacy, social and community context, healthcare access, and neighborhood and built environment. These findings extend previous research that has focused mainly on primary care physicians’ competencies (8), screening knowledge (14), and service provision (6) by illustrating how older adults evaluate the value of screening through the lens of their social resources, family responsibilities, and life experiences.

Concerns regarding economic burden and the lack of material incentives indicate that screening is often perceived as neither affordable nor offering immediate returns. Given the substantial family and socioeconomic burden of dementia in China (1), particularly costs associated with long-term care, medical treatment, and informal caring (28), older adults may regard screening not as an isolated test, but as the starting point of a potentially expensive care trajectory. Unclear follow-up pathways, insurance coverage, and care resources can transform an abnormal screening result into a perceived unaffordable future burden. Although dementia screening may be cost-effective at the population level, this does not necessarily translate into individual willingness to participate (29). Screening is more likely to be viewed as affordable and manageable when older adults understand how costs will be covered, how abnormal results will be managed, and how follow-up services will be coordinated. Modest material incentives may serve as a low-threshold trigger for initial engagement (30), particularly when health benefits are delayed and participation costs are immediate (31). Such incentives should complement, rather than replace, health education and must be embedded within standardized screening, result explanation, and follow-up management procedures to support sustained participation.

Older adults’ interpretations of cognitive risk were shaped by disease knowledge, educational attainment, and fixed beliefs about dementia risk. Consistent with prior research conducted in China (32), participants often demonstrated limited understanding of early dementia symptoms (33), risk factors, and intervention opportunities, and frequently normalized memory decline as a natural part of aging (34). Previous research among community-dwelling older adults has also suggested that greater dementia literacy may be associated with greater willingness to undergo dementia screening, although this relationship was not statistically significant (35). Importantly, our findings suggest that education may shape screening willingness through more complex pathways than a simple educational gradient. Limited knowledge among participants with lower educational attainment appeared to reduce recognition of the value of early screening, consistent with evidence linking dementia literacy with screening willingness (35). In contrast, some participants with higher education or professional backgrounds expressed greater confidence in their knowledge and judgment and therefore perceived less need for community-based screening. This apparent paradox suggests that greater knowledge does not necessarily translate into greater perceived need for professional assessment; confidence in one’s ability to interpret symptoms and judge personal risk may reduce the perceived added value of formal screening. Taken together, these findings challenge the assumption that educational attainment is a uniformly protective factor in screening decisions. Rather, education may shape not only access to health information but also how individuals interpret it in relation to their own cognitive vulnerability and the need for external professional judgment. This extends the relevance of health literacy beyond knowledge acquisition to the appraisal and application of health information in decisions about screening (36). Consistent with the Health Belief Model (37), some participants did not dismiss dementia as a serious condition but underestimated their own susceptibility and the potential benefits of screening. Self-assessment bias (38) may further reinforce reliance on personal judgement, particularly because early cognitive changes can be subtle. These findings suggest that community interventions should be tailored to the reasoning behind screening reluctance rather than to educational attainment alone. Alongside improving dementia knowledge, communication should help older adults better assess their personal risk and understand the added value of professional screening for early risk identification, functional preservation, and future care planning.

Perceived stigma, result-related anxiety and family concerns, and late-life values collectively revealed the social, familial, and life-stage meanings that older adults attach to screening results. Prior research has demonstrated that dementia-related stigma reduces help-seeking behavior (39), delays diagnosis and service use (40), and increases psychological distress among patients and their families (12). Our findings extend this evidence by showing that stigma operates prior to diagnosis, beginning at the screening stage. For older adults residing in close-knit communities, an abnormal result could signify diminished capacity, social judgement, and threats to personal identity, extending beyond health risk alone. Result-related anxiety and family concerns were closely intertwined in shaping screening avoidance. Possible abnormal findings evoked worry, fear, and images of future disability, making screening psychologically threatening. This aligns with information avoidance theory (41), which posits that individuals may avoid information they anticipate to be negative, uncontrollable, or difficult to manage. In this study, however, anxiety was not limited to personal fear of dementia; it was also tied to anticipated consequences for the family. Within the Chinese cultural context, older adults’ health decisions are strongly relational rather than driven solely by considerations of individual benefit. Filial piety and intergenerational responsibility profoundly shape dementia care in China (42), where family members often serve as primary caregivers (43) and the burden of care intersects with affiliate stigma (13). Some older adults expressed fear that an abnormal result would increase their children’s economic costs, caregiving responsibilities, emotional strain, and long-term obligations. These findings suggest that screening avoidance was shaped by anticipatory concerns encompassing both personal emotional distress and expected family caregiving consequences. Late-life values also influenced how screening was appraised. For some adults in advanced age, screening was considered meaningful only if it could improve quality of life, preserve dignity, or reduce family burden. This suggests that screening decisions in later life are not based solely on early detection or disease prevention, but also on whether screening is perceived as compatible with dignity, autonomy, and family responsibility. Community screening communication should therefore extend beyond early detection and delaying disease progression, and instead link screening to functional maintenance, reduced uncertainty, family preparedness, and the preservation of dignity in later life.

Insufficient medical trust and service assurance, limited information accessibility, and symptom-oriented healthcare-seeking behavior together created a rupture in the pathway from awareness to trust and subsequently to action regarding screening services. Prior studies have identified supply-side barriers to community-based dementia screening in China, including limited primary care capacity (9), unclear referral pathways (8), heavy workloads (44), and weak continuity of management (45). From the perspective of service users, this study further demonstrates that these supply-side deficiencies can translate into a trust gap among older adults. When community institutions appear insufficiently professional, results seem unreliable, or post-screening pathways remain unclear, screening is perceived as problem identification without practical resolution. Levesque et al. conceptualized healthcare access as extending beyond service availability to encompass individuals’ ability to perceive needs, seek care, reach services, pay for care, and engage with services over time (46). This study further indicates that, in the context of dementia screening, service trustworthiness and service linkage constitute additional core components of accessibility.

Limited information accessibility marked the starting point of this rupture. Some older adults had little prior exposure to dementia screening and were unaware of whether such services existed in their communities, leaving screening outside the scope of routine health decision-making and allowing memory decline to be interpreted as normal aging. Parker et al. reported that help-seeking for dementia diagnosis is persistently shaped by limited knowledge, stigma, family attitudes, and service accessibility (47). Jin et al.’s systematic review further demonstrated that dementia screening hesitancy is closely associated with disease awareness, expected benefits, psychological burden, and trust in services (48). Our findings are consistent with this evidence and further suggest that limited information does not merely represent a failure of publicity; rather, it constitutes an initial barrier that prevents older adults from progressing from risk perception to willingness to act. Concurrently, symptom-oriented healthcare-seeking behavior rendered this problem more pronounced. Many older adults tended to seek care only after noticeable symptoms or functional disruption had emerged, whereas early cognitive changes are often subtle, gradual, and not immediately disruptive to daily life. Community screening systems should therefore prioritize accessible, trustworthy, interpretable, and sustainable service pathways, positioning screening as an entry point for cognitive health management rather than a stand-alone activity.

Limitations in convenience and accessibility indicated that dementia screening was difficult to integrate into older adults’ everyday lives. Although dementia screening is frequently organized as a formal health service, participation depended on whether it was compatible with physical functioning, family responsibilities, schedules, and established daily routines. Harada et al. found that the distance to screening sites affects older adults’ participation in cognitive screening (49); Chen et al. similarly indicated that neighborhood and built environments are associated with cognitive health and dementia risk (50). This study further demonstrates that accessibility extends beyond geographic distance to encompass time costs, physical effort, waiting procedures, transportation convenience, caregiving responsibilities, and daily arrangements. For older adults of advanced age, those with mobility limitations, or those with spousal care and household responsibilities, even free and meaningful screening could be displaced by daily obligations when it required a separate trip and additional planning. Community screening should therefore be embedded within routine health examinations, chronic disease follow-up, family doctor services, community activities, home visits, and nursing follow-up, rather than relying on passive, attendance-based models.

This study reframes low participation in dementia screening among community-dwelling older adults as a behavioral dilemma shaped by social determinants, rather than a matter of individual attitude alone. Although prior research has examined dementia knowledge, screening attitudes (51), stigma, primary care capacity (48), and community management barriers (8), these factors are often investigated in isolation. Through the SDOH framework, our findings illustrate how these barriers accumulate across the screening pathway: economic instability undermines affordability; limited health literacy and biased self-judgment weaken perceived necessity; stigma, result-related anxiety, and family responsibilities magnify anticipated negative consequences; inadequate medical services, limited information, and symptom-oriented healthcare-seeking behavior diminish perceived benefits; and constraints within the neighborhood environment impede action. This layered mechanism provides empirical grounding for understanding why community-based dementia screening rates in China remain below policy targets (6, 7).

These findings suggest that community-based dementia screening should be designed as a coordinated service pathway, rather than as a stand-alone mobilization activity. Policy efforts should clarify the full screening pathway, including costs, insurance linkage, abnormal-result management, specialist referral, and long-term care arrangements. Health education should be tailored to older adults’ educational backgrounds and health beliefs, and should incorporate destigmatizing language along with robust privacy protection measures. Healthcare workers can serve as bridges between screening and ongoing care through risk communication, psychological support, family coordination, result interpretation, and follow-up linkage. Embedding screening within routine health examinations, chronic disease management, family doctor services, and home-based nursing care may help reduce the time, physical, and practical costs of participation.

Several limitations should be acknowledged. First, participants were recruited from three communities in Wuhan. Although purposive and maximum variation sampling was used to capture diverse perspectives across sociodemographic and SDOH contexts, the sample may not have fully captured the experiences of older adults facing greater socioeconomic or social disadvantage. The transferability of the findings to other populations, including migrant older adults, older adults living in rural areas, and nursing home residents may be limited. Second, the study focused exclusively on community-dwelling older adults and did not include family caregivers, community healthcare workers, or policy implementers, which limits insight into the interactions occurring across the screening implementation pathway. Finally, although the SDOH framework provided structure for data collection and analysis, its predefined nature may have constrained the identification of factors extending beyond the framework; inductive coding and team discussion were employed to mitigate this risk. Future multicenter mixed-methods studies should examine the relative contributions of different SDOH factors to both screening willingness and actual participation.

## 5 Conclusion

In summary, dementia screening among community-dwelling older adults is not hindered simply by insufficient knowledge, stigma, negative attitudes, or inadequate publicity, but by a complex behavioral dilemma shaped by economic stability, educational access and quality, social and community context, health care access and quality, and neighborhood and built environment. An SDOH-informed analysis shifts the understanding of screening from an individual-level issue to a system-level challenge. Future community screening programs should be grounded in older adults’ lived realities and supported by integrated systems that make costs affordable, information understandable, results explainable, services connected, and procedures convenient, enabling early dementia screening to become a sustainable community practice.

## Data Availability

The datasets presented in this article are not readily available because the datasets are not publicly available due to the risk of compromising participant privacy. De-identified data may be made available by the corresponding author upon reasonable request, subject to appropriate ethical and privacy considerations. Requests to access the datasets should be directed to Xianwu Luo, xwluo@whu.edu.cn.
