# Supplementary material for: Dilemmas of early dementia screening among community-dwelling older adults: a qualitative study from the perspective of social determinants of health
Source: Front Public Health. 2026 Sep 16;14:1927041. doi: 10.3389/fpubh.2026.1927041 (PMC13623565; doi:10.3389/fpubh.2026.1927041)
Supplement: Supplementary file 1 [file Table_1.DOCX]

Supplementary Materia1: Semi-structured Interview Guide

Title: Semi-structured interview guide for older adults

The interview guide was developed based on a review of relevant literature, the study aim, and the social determinants of health (SDOH) framework. The preliminary guide was reviewed by two nursing experts and one public health expert, pilot-tested with two older adults, and revised accordingly. The final guide was used to support and standardize the semi-structured interviews with community-dwelling older adults.

Before the interview, the interviewer introduced the study as follows:

Dear participant, we are members of a research team from the School of Nursing, Wuhan University. We are conducting a study on memory and early dementia screening among older adults in the community. We would like to ask you several questions. There are no right or wrong answers; please share your thoughts and experiences based on your own understanding and actual situation. With your permission, the interview will be audio-recorded to facilitate later data analysis. The audio recordings and transcripts will be used only for research purposes. All information will be kept confidential and securely stored, and your privacy will be protected.

The main interview questions were as follows:

1. What do you know about dementia?

2. Do you think it is necessary for older adults to undergo early dementia screening? Why or why not?

3. What benefits do you think participating in early dementia screening may bring to you?

4. What factors might prevent or discourage you from participating in early dementia screening? Please consider personal, family, and social factors.

5. How do you think a positive screening result might affect you and your family?

6. What are your views on the current screening methods, follow-up diagnosis, and treatment services provided by hospitals or community health services?

7. Do you have any other thoughts or suggestions regarding early dementia screening?
